# Effects of High-Intensity Functional Training (HIFT) on the Functional Capacity, Frailty, and Physical Condition of Older Adults with Mild Cognitive Impairment: A Blind Randomized Controlled Clinical Trial

**DOI:** 10.3390/life13051224

**Published:** 2023-05-21

**Authors:** Yulieth Rivas-Campo, Agustín Aibar-Almazán, Diego Fernando Afanador-Restrepo, Patricia Alexandra García-Garro, Gloria Cecilia Vega-Ávila, Carlos Rodríguez-López, Yolanda Castellote-Caballero, María del Carmen Carcelén-Fraile, María Leyre Lavilla-Lerma

**Affiliations:** 1 Faculty of Human and Social Sciences, University of San Buenaventura-Cali, Santiago de Cali 760016, Colombia; yrivasc@usbcali.edu.co; 2 Department of Health Sciences, Faculty of Health Sciences, University of Jaén, 23071 Jaén, Spain; mycastel@ujaen.es (Y.C.-C.); mccf0004@red.ujaen.es (M.d.C.C.-F.); llavilla@ujaen.es (M.L.L.-L.); 3 Faculty of Health Sciences and Sport, University Foundation of the Área Andina–Pereira, Pereira 660004, Colombia; dafanador4@areandina.edu.co; 4 Faculty of Distance and Virtual Education, Antonio José Camacho University Institution, Santiago de Cali 760016, Colombia; palexandragarcia@admon.uniajc.edu.co (P.A.G.-G.); gcvega@profesores.uniajc.edu.co (G.C.V.-Á.); 5 Lecturer University Schools Gimbernat, University of Cantabria, 39005 Santander, Spain; carlinhosdin@gmail.com

**Keywords:** physical activity, cognitive impairment, functional capacity, frailty, physical condition

## Abstract

Physical exercise has been established as an intervention in the integral approach for the physical, functional, and social health of older adults. The objective of this study was to determine the effects of a high-intensity functional training (HIFT) program on the physical condition and functional capacity of an elderly Colombian population with mild cognitive impairment. This research corresponds to a blind randomized controlled clinical trial. A total of 169 men and women aged over 65 years were evaluated and distributed in two groups: the experimental group that received a 12-week HIFT intervention (*n* = 82) and the control group (*n* = 87) that received general recommendations on the benefits of physical exercise. The outcome variables included physical condition, assessed using the Senior Fitness battery (SNB); Fried’s frailty phenotype was applied, and gait and balance were assessed using the Tinetti scale. For the functional variables, activities of daily living, instrumental activities of daily living, and advanced activities of daily living were evaluated. All variables were measured pre- and post-intervention. Statistically significant improvements were observed in the IG for gait stability and balance (*p* < 0.001), as well as for independence in activities of daily living (*p* = 0.003), and instrumental and advanced activities (*p* < 0.001). Likewise, greater functionality was found when assessed with the SNB (*p* < 0.001), except for upper limb strength. The frailty classification did not show changes post-intervention (*p* = 0.170) nor in the group x time interaction. MANCOVA analysis showed that regardless of gender, health level, age, BMI, cognition, and health level, the HIFT intervention produced better results in functional capacity, balance, and gait (*F* = 0.173, *p* < 0.001, Wilks’ *λ* = 88.231).

## 1. Introduction

In recent years, a considerable change in the dynamics of health has occurred due to the demographic and epidemiological transition that generated an increase in the population of older adults [1]. According to the World Health Organization (WHO), 125 million people over 80 years of age were reported in 2018, and it is estimated that by 2050, this will increase to 426 million people [2]. In Colombia, approximately 7 million people are older adults, equivalent to 14.4% of the country’s population, and by 2031, this number is expected to increase to 10 million [3]. These numbers highlight the challenges that will arise from this situation, such as the increase in the prevalence of cognitive impairment due to aging, as it is expected that the number of cases of dementia worldwide will grow from 57 million in 2019 to 153 million in 2050. Likewise in Colombia and other Latin American countries, an increase of 200% is expected [4].

Cognitive impairment often precedes dementia and may affect motor skills, leading to a gradual loss of independence in daily life, contributing to a poor quality of life [5]. The number of people with dementia continues to grow; however, there is still no cure, and the effects of pharmacological treatments are very limited [6]. Therefore, it is important to consider other types of treatments or interventions that could help to control the progression of dementia and delay any possible disabilities [6].

It has been estimated that approximately 8% of dementia cases could be prevented if all adults were physically active [7]; for this reason, programs that aim to increase physical activity levels could be a potential strategy to substantially reduce the burden of mild cognitive impairment and consequent dementia [7]. It is expected that in Colombia, about 23% of patients with mild cognitive impairment would be prevented if older adults perform vigorous physical exercise [8]. Additionally, physical exercise can preserve the ability to perform activities of daily living and functional skills in older adults [9,10]. An evidence-based rehabilitation program should start with a comprehensive assessment of the older adult and include therapeutic physical exercise, as well as other strategies that allow closer and more individualized management of frail patients, including patients with cognitive impairment, and consequently improve their quality of life and functionality [11,12].

An exercise modality that has gained great popularity in recent years is high-intensity functional training (HIFT) [13]. HIFT is characterized by using constantly varied high-intensity functional exercises that involve movements with body weight and/or external resistance [14]. Although HIFT has demonstrated great functional benefits [15,16,17], to our knowledge, no studies evaluating the effects of HIFT on the functional capacity, frailty, and physical condition of Colombian older adults with mild cognitive impairment have been published.

This research is of great importance due to the high social and economic burden that cognitive impairment during aging produces [18]. In addition, this research contributes to the decade of healthy aging in the Americas (2021–2030), aiming to improve the quality of life for the elderly in Latin America. Therefore, the objective of this study was to determine the effects of a HIFT program on the functional capacity, frailty, and physical condition of Colombian older adults with mild cognitive impairment.

## 2. Materials and Methods

### 2.1. Study Design

A randomized controlled clinical trial (NCT04638322) was carried out with pre- and post-intervention measurements, in which the participants were distributed using systematic randomization. This research was approved by the Human Ethics Committee of the University of Jaén.

### 2.2. Participants

The study participants were men and women over 65 years of age, with mild cognitive impairment, recruited from five geriatric centers in the city of Santiago de Cali, Colombia. The sample population was recruited using direct visits to the institutions where the project was presented, and the participants were able to decide on their participation. Those who met the following criteria were included in this study: (i) male and female users over 65 years of age, who voluntarily accepted to participate in this study, that did not participate in any additional physical exercise program; (ii) have sufficient physical autonomy to participate in the physical activities required by the study; (iii) have mild cognitive impairment (<25 in the Mini-Mental Status Examination); and (iv) being able to understand the instructions, programs, and protocols of this trial. The following subjects were excluded: (i) persons with medical advice to avoid physical exercise; (ii) persons diagnosed with cancer, pulmonary hypertension, renal failure, heart failure, and/or any orphan diseases; (iii) persons under psychiatric treatment or with neurological alterations; (iv) persons using beta-blockers medication; (v) persons infected with the human immunodeficiency virus (HIV/AIDS); and (vi) persons that did not accept to participate in this study or, at the moment of entering the program, refused to accept the use of their data for research outlined in the informed consent form.

### 2.3. Intervention

Group assignment was randomly sampled using the Epidat 3.1 program (Xunta de Galicia. Consellería de Sanidade-Servizo Galego de Saúde), and the allocation was concealed. The assignment was performed by a researcher who did not intervene in the subsequent phases of assessment, intervention, data recording, or database analysis. The persons who carried out the pre- and post-intervention assessments were trained in the use of the tests and did not participate in the intervention nor communicate with the persons who conducted the HIFT program. The analysis of the results was carried out by a different researcher. In addition, the assessors and the performer of the statistical analysis were blinded.

#### 2.3.1. Control Group (CG)

The control group was not exposed to any additional intervention. Participants assigned to this group received general advice on the positive effects of regular physical activity and were given the PAHO physical activity recommendations guide. Outcome variables were collected at baseline and at the end of this study.

#### 2.3.2. Intervention Group (IG)

The intervention protocol consisted of a HIFT program with exercises aimed at a basic level with a length of 12 weeks, a frequency of 3 sessions per week, and duration of 45 min each. The exercise program had three phases: first, a 10 min warm-up composed mainly of joint mobility exercises; second, a 25 min core phase divided into 4 intervals, in which participants performed exercises at an 80–85% intensity of their maximum heart rate that included bicycle-like limb movements from a seated position, wall push-ups in a standing position, chair squats, and ball throws against the wall while performing lateral and front lunges. Each exercise was performed for 30 s and as fast as possible avoiding any impact on the joints; then, the subject rested for 15 s before repeating the exercise. Each work interval had a duration of 4 min with an active rest for 3 min at 50–70% of the maximum heart rate that included lateral walking activities alternating with heel raises, lateral and frontal upper limb raises, and functional diagonal reaches with trunk rotation. Finally, there was a 10 min cool down with muscle stretching and relaxation and breathing techniques.

Heart rate was monitored with pulse sensors (Polar RS300Xsd) placed on the wrist of each participant. The trainer who led the activity had the logistical support of nursing assistants or trained persons who were assigned to the participants. Each assistant was assigned to a maximum of 2 subjects, who were supervised both during the workout and rest phase to ensure that the required intensity was achieved.

### 2.4. Outcome Measurements

The variables used in this research were collected by the assessment team, which were the same for both the CG and the IG. Sociodemographic data such as age, sex, socioeconomic status, schooling, and marital status were collected. Anthropometric variables were also obtained, including weight (direct measurement using a precision scale from 100 g to 120 kg, Kenwel Dt612^®^ Omagh, North Ireland), height (precision measuring scale from 1 mm, SECA 213), and body mass index (BMI), which was obtained as the weight of each participant in kilograms divided by their height in square meters. Clinical characterization variables were recorded at baseline as the Charlson health condition and cognitive level with the Mini-Mental Status Examination (MMSE). This research focused on measuring physical condition and functional capacity, addressing conditions of frailty and independence.

#### 2.4.1. Physical Condition and Functional Capacity

For the physical condition and functional capacity variables, the Senior Fitness Battery (SFB) [19] was used. The SFB consists of the following tests: the arm curl and chair stand test for upper and lower limb muscular strength, respectively; the 6-min walk test to evaluate aerobic capacity; the chair sit-and-reach test for lower limb flexibility; the back scratch test for upper limb flexibility; and the 8-foot up-and-go test to evaluate agility and dynamic balance. This battery was designed by Rikli and Jones and is widely used in studies on older adults [20].

#### 2.4.2. Gait and Balance

The Tinetti scale assesses postural stability, balance, and gait, which allows a health professional to determine a patient’s risk of falling. The assessment is divided into two parts: the first assesses balance and the second assesses gait. The maximum score for balance is 16, and for gait, it is 12. From the sum of both, a maximum score of 28 is obtained. This score was used to classify the risk of falls, where it was considered that with a total score between 19 and 24, the risk of falls is minimal, while a total score < 19 indicates a high risk of falls. The Tinetti scale is valid and reliable for use in Colombian older adults [21].

#### 2.4.3. Frailty

Frailty was assessed using the frailty phenotype [22], which consists of five criteria: (i) unintentional weight loss; (ii) self-reported exhaustion; (iii) slow walking speed; (iv) weakness; and (v) low physical activity. If an older adult presented >3 criteria, he/she was classified as frail; if only 1 or 2 criteria were presented, he/she was classified as moderate frail; and he/she was classified as non-frail when no criteria were observed.

#### 2.4.4. Functional Capacity and Independence

The Katz index examines activities of daily living (ADL) such as bathing, dressing, toileting, transferring, continence, and feeding, for which dependence or independence to perform these activities were considered. It was designed to be used on people over 65 years of age and has been validated for universal applicability [23].

On the other hand, the Lawton and Brody index evaluates instrumental activities of daily living (IADL) such as shopping, cooking, cleaning, washing, finances, medication, transportation, and telephone use. It considers instrumental disability as the inability to perform one or more activities [24] and has been validated in Spanish for the geriatric population [25].

Finally, the Siu and Reuben Physical Scale for Advanced Activities of Daily Living (AAVD) assesses the degree of integration and social relationships in the older adult [26].

### 2.5. Sample Size Calculation

The sample size was determined using the freely available statistical software Epidat 3.1 (Xunta de Galicia. Consellería de Sanidade-Servizo Galego de Saúde) with the following parameters: a confidence level of 95%, a significance level of 5%, a power of 90%, and an expected proportion of improvement of 30% in the IG vs. 15% in the CG, resulting in a total of 132 persons required (66 participants per group). This value was adjusted to an expected loss percentage of 15%, obtaining a final required sample of 152 persons distributed in 2 balanced groups with a minimum of 76 participants. The sampling was performed randomly using the same statistical program.

### 2.6. Statistical Analysis

An exploratory analysis was performed using the data obtained, identifying the normality of the data distribution using the Kolmogorov–Smirnov test (*p* > 0.05). From the univariate analysis, we proceeded to characterize the study population by presenting the sociodemographic variables and outcome variables for each group: CG and IG. The quantitative variables were presented as the mean value and its standard deviation (SD) given the normality test result. Qualitative variables were presented as the frequency and percentage in each category. To test the comparability between groups, statistical methods such as chi-square were used for categorical variables and the *t*-test for quantitative variables.

For the analysis of the variables related to the intervention, a mixed analysis of variance (ANOVA) was used, with the between-group factor being participation or not in the HIFT program and the within-subjects factor being the time of measurement. Cohen’s *d* was used to calculate intergroup effect sizes, where a value of ≤0.2 indicated a small effect, <0.8 a medium effect, and ≥0.8 a large effect. This analysis allowed the determination of whether there were significant differences between the groups in this study depending on the time at which the measurements were taken. In addition, it allowed the analysis of whether there were significant interactions between the group and time factors. A multiple model integrating all the quantitative outcome variables with analysis of covariance (MANCOVA) was performed to evaluate the influence of the independent variables (age, sex, BMI, MMSE, and health condition) followed by univariate F-tests using Wilks’ λ statistic. The categorical variables were analyzed using a multinomial and binomial multiple analysis appropriately adjusted for age, sex, BMI, MMSE, and health condition. For all statistical tests of hypothesis contrasts, a significance level of 0.05 and a reliability level of 95% were established. All statistics were performed with the statistical package Stata 14.0.

## 3. Results

**Figure 1:**
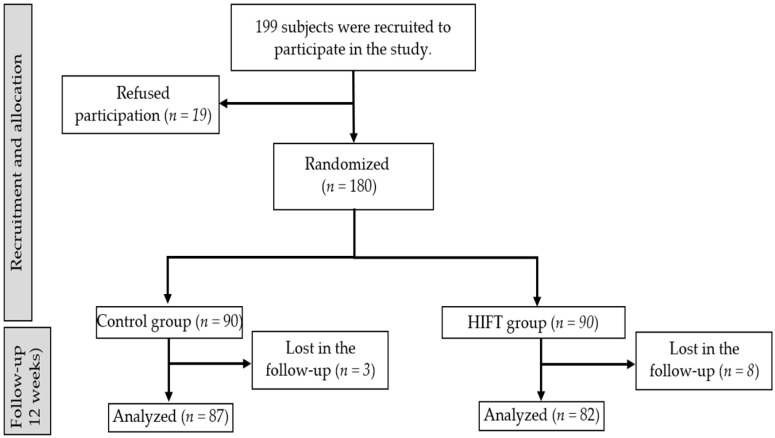
CONSORT flow diagram showing participant selection and allocation.

A total of 257 persons were considered, of whom 199 met the inclusion criteria. Overall, 180 persons were recruited from elderly care institutions and randomized to a CG or IG, and 169 of them remained in the study until the end, of which 66 were men and 103 were women (Figure 1).

The participants completed 95.5% of the total sessions, showing high adherence to the intervention. A sociodemographic description of the participants and their baseline measures are presented in Table 1, which shows that no significant differences were found between the characteristics of the groups before starting the intervention. The study population belongs to the middle socioeconomic level (73.4%), most of them had a high school education (56.2%), were married (51.5%), and presented right-handedness (95.3%). There were no reports of adverse events during the course of this study.

### 3.1. Functional Capacity

The statistical analysis showed differences between the CG and the IG in the post-intervention measurements, favoring the HIFT group (Table 2). Better results were shown for lower body strength in the chair stand test (*p* = 0.049) with a small effect size (Cohen’s *d* = 0.307), as well as improvement in upper body flexibility in the back scratch test (*p* < 0.001) with a medium effect size (Cohen’s *d* = −0.573). A significant and large effect size was observed for gait in the 6-min walk test (*p* < 0.001; Cohen’s *d* = 1.305) and lower body flexibility in the chair sit-and-reach test (*p* < 0.001; Cohen’s *d* = 1.120), while in the arm curl, test no differences between groups were shown (*p* = 0.217). The intragroup analysis showed changes in the IG for all the variables of the SFB except for the arm test (T = −1.060, *p* = 0.291).

Taking into account the mixed variance Group × Time, it was determined that there were significant differences between the treatment groups as a function of time showing changes in the IG for the following tests: the chair stand test (*F* = 14.70, *p* = 0.032, *η*^2^ = 0.014), 6-min walk test (*F* = 49.6, *p* < 0.001, *η*^2^ *=* 0.093), chair sit-and-reach test (*F* = 41.00; *p* < 0.001, *η*^2^ = 0.064), back scratch test (*F* = 22.95, *p* < 0.001, *η*^2^ = 0.029), and 8-foot up-and-go test (*F* = 24.22, *p* < 0.001, *η*^2^ = 0.23). Regarding upper body strength (arm curl test), no changes were found between groups (*p* = 0.290) nor in the Group × Time interaction (*F* = 1.56, *p* = 0.213) (Table 2). These results confirm that after the intervention with HIFT, an improvement was found in the functionality evaluated with the SFB (*p* < 0.001) except for upper limb strength.

A multiple analysis (MANCOVA) was performed, which showed that when evaluating all the quantitative outcome variables (functional capacity, balance, and gait) and their interaction with the independent variables (sex, health level, age, BMI, cognition, and health level), the effect of the HIFT intervention was maintained for most of the variables (*F* = 0.173, *p* < 0.001, Wilks’ *λ* = 88.231). However, the influence of age was found in the outcomes associated with gait (8-foot up-and-go test and Tinetti *p* < 0.001) (Table 3).

### 3.2. Gait and Balance

Regarding the Tinetti scale result, the Group × Time analysis showed differences in the group as a function of time, finding higher scores in the IG for both the balance (*F* = 36.01, *p* < 0.001, *η*^2^ = 0.057) and the gait (*F* = 97.0, *p* < 0.001, *η*^2^ = 0.042) components of the Tinetti scale. These values indicate that the IG presented a higher degree of postural stability; an ability to hold different positions, including standing with eyes open or closed; an ability to change position without losing balance; a greater patient walking ability; symmetry in their steps; and an increased ability to perform tasks such as turning and changing direction while walking.

### 3.3. Fall Risk

In addition, the Tinetti scale was analyzed categorically to identify the risk of falls and the change in this risk for the groups. At baseline, it was observed that a higher percentage of participants were at minimal risk of falling (CG = 52.9% and IG = 47.6%) with no differences between groups (*p* = 0.483). However, at the end of the intervention, these values were modified, showing that the CG increased its minimum risk of falling to 56.3%, while the IG reached 61%, with a significant intragroup (*p* = 0.020) and intergroup (*p* = 0.002) difference. The multinomial regression model (Table 4) showed that regardless of age, sex, BMI, global cognition score, and health condition presented by the participants, the odds of changing from a high-level risk of falls to a minimal-risk level in the IG was 2.59 times greater than in the CG (*p* = 0.015) and 6.9 times (*p* < 0.001) that of reaching the no-risk level. These results indicate that the IG showed a greater decrease in the risk of falls when compared to the CG.

### 3.4. Frailty

At baseline, both groups presented a higher percentage of the population in the frailty condition according to Fried’s phenotype (CG = 55.2% and IG = 58.5%), confirming equal conditions at baseline (*χ*^2^ = 2.26, *p* = 0.320). Although an intragroup difference was found in the population treated with HIFT at the end of the intervention, the comparison between groups identified that the levels of frailty remained without significant differences (*χ*^2^ = 6.39, *p* = 0.170), ruling out the influence of HIFT on the frailty condition.

The Group × Time interaction analysis determined that the predictor Group was not significant for either comparison. Specifically, compared to the “Non-frail” reference group, moderate frailty was positively associated with a constant of 0.68, suggesting that moderate frailty has an expected value of 0.68 units higher than the CG group. The predictor Time was not significant (*p* = 0.670) in this comparison, indicating that changes over time (pre- and post-test) were not significantly related to frailty. Compared to the Non-Frail and Frailty categories, it was evident that the Time and Group predictors were not significant for this comparison, indicating that neither changes over time nor the treatment group are significantly related (*p* = 0.260). These results show that frailty remained the same in both groups, which suggested no influence of time and group on the variable.

Finally, the multinomial model confirmed that regardless of the sociodemographic and clinical variables, the IG and the CG did not show significant differences with respect to the opportunity to change levels of frailty (Table 4).

### 3.5. Independence

The baseline values showed that the percentage of the population classified as without dependence according to the Katz index was 43.8% in the CG and 52.4% in the IG (*p* = 0.475). After the intervention, the CG had no intragroup change (43.7%), but a significant increase was observed in the IG (69.5%, *p* = 0.001), which showed significant differences between groups in executing activities of daily living in favor of the post-treatment IG (*p* = 0.003). The interaction analysis indicated that the changes in the categories of mild and moderate dependence with respect to the without-dependence category were significantly related to the group and time factors (*p* = 0.007 and *p* = 0.003 respectively) (Table 3).

After adjusting for age, sex, BMI, MMSE, and health condition, the multinomial model indicated that the group that underwent HIFT training had 16 times the odds of changing their condition from moderate dependence to without dependence in ADL when compared with the CG (*p* < 0.001) (Table 4).

The assessment of IADL based on the Lawton and Brody scale showed that the population under study presented mild to moderate dependence, with the first category having the highest proportion in both the CG (83.9%) and IG (85.4%). In the post-intervention measurements, although the highest proportion remained in the mild dependence category (CG = 74.7% and IG = 100%), it was observed that in the CG, there was a significant change that showed an increase in the number of people in the moderate dependence category (25.3%), while the IG managed to reduce the number of people with moderate dependence to 0%. The change in the distribution of percentages between groups at the end of treatment was significant and ratified a lower level of dependence after treatment with HIFT (*p* < 0.001). Likewise, the interaction analysis showed that changes in time and the treatment group were significantly related to the level of dependence in IADL (*p* < 0.001) (Table 3).

The binomial regression analysis used the moderate dependence level as a starting point and showed that in the group that performed the HIFT program, the odds of change toward the mild-dependence level in the IADL is 1.5 times that for the CG; however, the significance decreased when adjusting for age, BMI, MMSE, and health condition (*p* = 0.05) (Table 4).

From the baseline measurement of the Siu and Reubens scale, the lack of autonomy in advanced activities of daily living prevailed in both groups (CG = 78.2% and IG = 67.1%); however, after the intervention, a decrease in these percentages was observed (CG = 75.9% and IG = 46.3%). The intragroup analysis showed significant changes (pre–post) in the IG that favored autonomy and demonstrated statistically significant differences between groups (*p* < 0.001). Likewise, the Group × Time interaction analysis was significant (*p* < 0.001). The binomial multiple regression analysis (Table 4) evaluated the opportunity to become autonomous, and it confirmed that the IG had 1.9 times the odds of the CG to demonstrate this progress regardless of the level of MMSE, BMI, and health condition (*p* = 0.042); however, this process can be influenced by age, favoring the youngest (*p* < 0.001).

Based on these results, it can be stated that the HIFT intervention significantly improved the ability to perform activities of daily living. Additionally, a significant decrease in dependence for advanced activities of daily living and an improvement in autonomy were also observed in the IG.

## 4. Discussion

The objective of this study was to determine the effects of a 12-week HIFT program on the physical condition, frailty, and functional capacity of Colombian older adults with mild cognitive impairment. Hence, the main findings of this study indicate significant improvements in aerobic capacity, lower body strength, flexibility, functional capacity, and postural stability, as well as a decrease in the risk of falls due to the intervention.

In this study, the HIFT program had a structure with high-intensity intervals at 80–85% of the maximum heart rate (HRmax), which were similar to a previous study that reported an intensity at 75–85% of the peak oxygen volume (VO2peak) [27]; however, the intensity used in our study was lower than the intensity used by several studies reviewed by Marriott et al. [28], where various types of training were used, as well as various ways to measure exercise intensity, i.e., 18 points on the Borg scale; 85–95% of the peak oxygen volume (VO2max); 90–95% HRmax; and 90–110% of the peak power output (Wpeak). This highlights the great methodological diversity used in high-intensity exercise interventions.

In addition, it is important to emphasize that our findings with regard to the improvement in aerobic capacity are consistent with those from other studies in which high-intensity intervallic training compared to moderate-intensity intervallic training and/or continuous training more effectively improved cardiovascular and respiratory function variables in older adults [29,30] as well as in other populations [31], even in persons with cardiac disease [32]. Apparently, the improvement in some variables such as maximal mitochondrial respiration in muscle fibers, PGC-1α content, p53, and PHF20 seem to be due to the increase in the intensity and not the type of exercise. This was proven by analyzing two cycloergometer protocols for intervallic training: sprints at ∼200% of Wpeak compared with intervals at ∼90% of Wpeak [33]. Similarly, aerobic capacity assessed with the 6-min walking test improves due to the increase in intensity regardless of the type of exercise, as the improvement is observable with fast walking/running, high-intensity interval training (HIIT) protocols with gradient changes [29], and with functional exercises such as those proposed in our study, as well as by other authors [34].

Additionally, our intervention improved lower body strength, which could be due to high metabolic stress caused by the time under tension that HIFT interventions produce when performing a high number of repetitions at maximum speed with short rests [17]. This structure is similar to that used in acute HIFT sessions, where increased levels of interleukin-6 (IL-6) and blood lactate were observed after exercise [35], favoring muscle adaptations related to muscle growth [36,37,38,39]. This is consistent with other interventions where functional training improved components of strength in older adults [40,41].

However, not all HIIT-based interventions have similar effects on lower limb strength, as evaluated with the 30 s chair stand test. Aboarrage et al. [42] reported improvements in strength following 24 weeks of high-intensity aquatic training (HIAT), based on 30 s jumping intervals, at maximal intensity (single leg hopping, ankle hopping, shrugging, jumping with hip abduction and adduction), while Bruseghini et al. [43] stated that eight weeks of 2-min intervals on a cycloergometer at 85–95% of the VO_2_max did not improve strength levels in older adults. This suggests that the type of exercise could be an important modulator of strength gains. Moreover, according to a recent meta-analysis, HIFT seems to represent an appropriate method for inducing chronic improvements in motor functions [44].

On the other hand, after our intervention with HIFT, no improvements in upper limb muscle strength/endurance were observed. This might be because the exercises used in this intervention (flexion of the arms on the wall from a standing position) did not achieve an adequate stimulus in the musculature. It is important to highlight that improvement in the physical condition is a fundamental objective in any exercise program, given the implications that an adequate or deficient physical condition can have on various aspects of health. For example, it is known that older adults who are in the early stage of fall risk tend to have a lower physical condition [45].

The HIFT intervention improved postural stability and decreased the risk of falls; this may be due to a combined effect among (1) the biomechanical stimulation generated with exercises that shift the center of gravity and modify the base of support (in this research: lateral lunges, squats, and static standing positions with legs apart and legs together). This training strategy has been used in different exercise interventions with positive effects on stability in older adults [46]. The evidence suggests that it also improves balance in older adults with a record of falls [47]. (2) An increase in lower limb strength, which is consistent with previous studies that indicate a negative association between strength and fall risk. In addition, it has been reported that an improvement in lower limb strength is followed by an improvement in stability in older people [48]; however, a previous study on stability and risk of falls showed that moderate-intensity strength training (50–75% of 1 RM) with weight machines, which included various upper and lower limb exercises, decreased the risk of falls but did not improve stability in older adults [49]. (3) The stimulus produced with high-intensity exercise: it has been reported that intervention with HIIT improved stability and decreased the risk of falls in older adults [50], which is consistent with the results of an intervention based on a HIFT program that improved stability in older adults with dementia [51].

Moreover, it is known that a low level of physical activity represents one of the modifiable factors related to the severity of frailty in the aging [52]. Although the severity of this condition can change due to interventions based on exercise [53,54], the results of the present study suggested that HIFT has no influence on the frailty condition when assessed with Fried’s frailty phenotype. This could be explained by the short duration of the intervention since the effects on frailty appear to be achieved with a more prolonged intervention of 23 weeks [55]. Another factor that determines the improvement in frailty is the implementation of stimuli that promote the development of muscle strength, as has been previously demonstrated [56,57,58]; however, the exercises used in this study were insufficient for this purpose.

Additionally, it should be considered that the frailty syndrome is multidimensional, characterized by a reduction in functional capacity and/or in the ability to deal with different types of stressors [59]. Several dimensions of frailty have been identified such as emotional frailty [60], social frailty [61], and physical frailty [22]. Therefore, in addition to the results obtained with Fried’s phenotype, the changes induced with HIFT training on physical condition and functional capacity, which are related to physical frailty, should be considered [59,62]. Hence, it can be inferred that HIFT training improved specific aspects of physical frailty.

Furthermore, the ability to perform the activities necessary to live independently represents an important objective in the exercise programs oriented to this population [63] since is essential to maintaining health well-being and the quality of life during aging [64,65,66]. We found that as a result of the intervention using HIFT, older adults with moderate dependence have 16 times the opportunity to change their condition to no dependence in ADL. In addition, improvements in the IADL assessment and autonomy in AADL were expected, since it has been reported that a higher level of physical activity is associated with improvements in the functional capacity of older adults [67]. However, it is important to establish the volume of training necessary to obtain desirable results. In this sense, and similar to our research, Ramos et al. [68], reported that circuit resistance training is also effective in significantly improving the functional capacity of older adults.

The absence of falls, pain associated with the intervention, and cardiovascular or osteoarticular accidents among the participants who underwent the exercise intervention suggests that the HIFT program does not represent an additional risk for this population. When added to the effectiveness of this intervention on the improvement in the physical condition and functional capacity of older adults, this allows proposing that the HIFT program is a promising alternative with great potential to help manage the evolution and slow the progression of cognitive impairment in older adults [6]. Therefore, this type of physical training intervention could relieve the negative impact of this disease on the quality of life of older adults and also on the socio-economic burden [69]. It is important to emphasize that further follow-up studies are needed to clarify the impact of this type of training on physical and functional capacities and on the preservation of cognitive function in the long term.

Finally, despite the good results obtained after the intervention, we identified some limitations such as not having evaluated the level of acceptance or enjoyment generated with this intervention, which was reported in a recent study on the feasibility and efficacy of a HIFT protocol, where the rate of adherence, acceptability, and effectiveness of this type of intervention was encouraging [70]. Likewise, given that the HIFT-based intervention was not compared with another intervention based on traditional or moderate-intensity exercise, the results should be interpreted with caution. Finally, the dimensions of frailty status were not analyzed in an independent manner, which could have helped to clarify the supposed harmless effects of this program on frailty status.

## 5. Conclusions

From the results obtained, it is possible to state that a twelve-week program of high-intensity functional training is safe for Colombian older adults with mild cognitive impairment, in addition to significantly increasing their physical condition and functional capacity, which also improves physical frailty in this population. These results highlight the importance of designing and implementing HIFT-based programs oriented to older people with this condition, generating an important impact not only on the quality of life of the subjects but also on the economic burden that these patients represent to the health system. The results of this research could be useful in the design of evidence-based exercise guidelines and public policies aimed at improving the well-being of the population.

## Figures and Tables

**Table 1:** Sociodemographic characteristics at baseline.

		Total (n = 169)	CG (n = 87)	IG (n = 82)	*p* Value
Age. Mean (SD)		77.1 (7.41)	76.8 (7.4)	77.4 (7.3)	0.616
MMSE. Mean (SD)		21.6 (1.4)	21.1 (1.2)	21.5 (1.5)	0.689
BMI. Mean (SD)		27.9 (4.7)	27.8 (5)	28.1 (4.2)	0.657
Sex. n (%)	Female	103 (60.9)	53 (60.9)	50 (61)	0.990
	Male	66 (39.1)	34 (39.1)	32 (39.0)	
Socioeconomic strata. n (%)	1	0 (0.0)	0 (0.0)	0 (0.0)	0.884
	2	0 (0.0)	0 (0.0)	0 (0.0)	
	3	53 (31.4)	29 (33.3)	24 (29.3)	
	4	71 (42.0)	34 (39.1)	37 (45.1)	
	5	28 (16.6)	15 (17.2)	13 (15.8)	
	6	17 (10)	9 (10.3)	8 (9.8)	
Education level. n (%)	Elementary school	25 (14.8)	14 (16.1)	11 (13.4)	0.820
	High school	95 (56.2)	47 (54.0)	48 (58.5)	
	College	46 (27.2)	25 (28.7)	21 (25.6)	
	Postgraduate	3 (1.8)	1 (1.1)	2 (2.4)	
Marital status. n (%)	Single	20 (11.8)	10 (11.5)	10 (12.2)	0.411
	Married	87 (51.5)	48 (55.1)	39 (47.5)	
	Divorced	18 (10.7)	6 (6.9)	12 (14.6)	
	Widowed	44 (26.0)	23 (26.4)	21 (25.6)	
Laterality. n (%)	Right	161 (95.3)	83 (95.4)	78 (95.1)	0.562
	Left	7 (4.1)	4 (4.6)	3 (3.6)	
	Both	1 (0.6)	0 (0.0)	1 (1.2)	
Health status. n (%)	High	108 (63.9)	55 (63.2)	53 (64.6)	0.848
	Low	61 (36.1)	32 (36.8)	29 (35.4)	

SD: Standard Deviation; MMSE: Mini-Mental Status Examination; CG: Control Group; IG: Intervention Group; n: Frequency.

**Table 2:** Effects of HIFT on physical condition, functional capacity, gait, balance, frailty, functionality, and independence.

Quantitative Outcomes		Pre			Post			Group			Time			Group × Time		
		CG	IG	*p*-Value	CG	IG	*p* Value	*F*	*P*-Value	*η* ^2^	*F*	*p*-Value	*η* ^2^	*F*	*p*-Value	*η* ^2^
Chair Stand Test. Mean (SD)		9.7 (2.0)	9.4 (2.3)	0.342	9.7 (7.7)	11.5 (2.0)	0.490	2.41	0.123	0.070	4.81	**0.03**	0.014	4.70	**0.032**	0.014
Arm Curl Test. Mean (SD)		11.3 (2.6)	11.1 (2.8)	0.751	10.2 (2.1)	10.8 (2.5)	0.217	1.51	0.221	0.004	1.21	0.274	0.004	1.56	0.213	0.005
Six-Minute Walk Test. Mean (SD)		387.4 (49.2)	389.8 (53.0)	0.759	386.0 (67.5)	475.1 (69.0)	**<0.001**	43.42	**<0.001**	1.040	46.50	**<0.001**	0.087	49.60	**<0.001**	0.093
Chair Sit-and-Reach Test. Mean (SD)		−5.1 (1.8)	−5.1 (1.6)	0.947	−5.9 (1.7)	−4.0 (1.7)	**<0.001**	17.81	**<0.001**	0.067	1.21	0.272	0.002	41.00	**<0.001**	0.064
Back Scratch Test. Mean (SD)		−4.8 (1.7)	−5.0 (1.6)	0.445	−5.1 (1.7)	−4.1 (1.6)	**<0.001**	2.84	0.094	0.010	7.79	**0.006**	0.010	22.95	**<0.001**	0.029
Eight-Foot Up-and-Go Test. Mean (SD)		9.6 (2.5)	9.5 (2.1)	0.845	11.4 (2.9)	4.6 (1.9)	**<0.001**	89.72	**<0.001**	0.240	5.16	**<0.001**	0.050	24.22	**<0.001**	0.230
Tinetti Gait. Mean (SD)		9.5 (1.6)	10.0 (1.6)	0.850	9.3 (1.6)	10.4 (1.4)	**<0.001**	10.80	**0.001**	0.057	3.52	0.062	0.001	36.01	**<0.001**	0.011
Tinetti Balance. Mean (SD)		11.0 (2.2)	10.8 (2.3)	0.663	10.5 (2.1)	19.8 (3.3)	**<0.001**	5.73	**0.018**	0.029	29.8	**<0.001**	0.013	97.00	**<0.001**	0.042
Tinetti Total. Mean (SD)		20.4 (3.4)	20.7 (3.8)	0.521	19.8 (3.3)	22.6 (2.9)	**<0.001**	9.54	**0.002**	0.049	28.4	**<0.001**	0.009	94.4	**<0.001**	0.029
**Categorical Outcomes**		**Pre**			**Post**			**Group**			**Time**			**Group × Time**		
		**CG**	**IG**	***p* value**	**CG**	**IG**	***p* value**	**Odds**	**IC 95%**	***R* ^2^**	**Odds**	**IC 95%**	***R* ^2^**	**Odds**	**IC 95%**	***R* ^2^**
Tinetti. *n* (%)	Without risk	12 (13.8)	17 (20.7)	0.483	8 (9.2)	20 (24.4)	**0.002**	Reference		0.01	Reference		0.004	Reference		0.010
	Minimum risk	46 (52.9)	39 (47.6)		49 (56.3)	50 (61.0)		0.68	(0.60 to 0.76)		−0.188	(−0.19 to 1.01)		−1.463	(−1.49 to −1.40)	
	High risk	29 (33.3)	26 (31.7)		30 (34.5)	12 (14.6)		−0.37	(−0.31 to 1.08)		−0.435	(−0.45 to 1.09)		−0.269	(−0.33 to 1.07)	
Frailty. *n* (%)	Non-Frail	14 (16.1)	7 (8.5)	0.320	14 (16.1)	15 (18.3)	0.170	Reference		0.009	Reference		0.006	Reference		0.002
	Moderate Frail	25 (28.7)	27 (32.9)		26 (29.9)	36 (43.9)		5.92	(0.99 to 6.2)		0.907	(0.86 to 1.75)		0.68	(0.67 to 1.06)	
	Frail	48 (55.2)	48 (58.5)		47 (54.0)	31 (37.8)		1.22	(1.18 to 1.25)		2.190	(0.89 to 2.66)		1.42	(1.39 to 1.45)	
Katz Index. *n* (%)	Without dependence	38 (43.8)	43 (52.4)	0.475	38 (43.7)	57 (69.5)	**0.003**	Reference		0.010	Reference		0.021	Reference		0.020
	Mild dependence	36 (41.4)	27 (32.9)		30 (34.5)	17 (20.7)		−0.68	(−0.71 to −0.61)		−4.524	(−4.90 to 1.36)		−0.855	(−0.95 to −0.89)	
	Moderate dependence	13 (14.9)	12 (14.6)		19 (21.8)	8 (9.8)		−0.74	(−0.81 to−0.67)		−0.825	(−0.99 to 1.27))		−0.8151	(−0.86 to −0.80).	
Lawton and Brody Index. *n* (%)	Mild dependence	73 (83.9)	70 (85.4)	0.793	65 (74.7)	82 (100.0)	**<0.001**	Reference		0.004	Reference		0.004	Reference		0.004
	Moderate dependence	14 (16.1)	12 (14.6)		22 (25.3)	0 (0.0)		1.20	(1.93 to 1.28)		0.195	(0.192 to 0.199)		1.197	(1.14 to 1.22)	
Siu and Reubens Scale. *n* (%)	Autonomous	19 (21.8)	27 (32.9)	0.106	21 (24.1)	44 (53.7)	**<0.001**	Reference		0.030	Reference		0.010	Reference		0.040
	Not autonomous	68 (78.2)	55 (67.1)		66 (75.9)	38 (46.3)		0.93	(0.90 to 0.95)		0.501	(0.43 to 0.62)		0.954	(0.93 to 0.96)	

SD: Standard Deviation; n: Frequency; CG: Control Group; IG: Intervention Group.

**Table 3:** Outcomes included in the MANCOVA: functional capacity, balance, and gait in interaction with sex, health condition, age, BMI, and cognition.

	Wilks’ Lambda Value	F	*p*-Value
Group	0.173	88.231	**<0.001**
Charlson	0.965	0.661	0.725
Sex	0.956	0.845	0.564
Group × Charlson	0.973	0.513	0.845
Group × Sex	0.942	1.141	0.339
Charlson × Sex	0.957	0.841	0.568
Group × Charlson × Sex	0.960	0.770	0.630
Age	0.539	15.826	**<0.001**
BMI	0.967	0.631	0.750
MMSE	0.971	0.556	0.812

Charlson: Health condition; BMI: Body Mass Index; MMSE: Mini-Mental Status Examination.

**Table 4:** Post-intervention multinomial regression for fall risk, frailty, independence in ADL, IADL, and AADL adjusted for age, sex, BMI, MMSE, and health condition.

RISK OF FALLING *	Multinomial Model	*p*-Value	Odds Ratio	*η* ^2^
Minimum	Group: IG–CG	0.015	2.60	0.082
Without risk	Group: IG–CG	<0.001	6.95	
FRAILTY *	Multinomial Model	*p*-value	Odds ratio	
Moderate Frail	Group: IG–CG	0.130	2.53	0.252
Non-Frail	Group: IG–CG	0.760	2.68	
INDEPENDENCE ON ADL *	Multinomial Model	*p* value	Odds ratio	
Without dependence	Group: IG–CG	<0.001	16.69	0.289
Mild dependence	Group: IG–CG	0.760	3.06	
IADL **	Binomial Model	*p*-value	Odds Ratio	
Age		<0.001	0.61	
MMSE		0.381	0.81	0.319
BMI		0.69	0.97	
Group: IG–CG		0.054	1.51	
Health condition: low–high		0.696	0.73	
AADL ***	Binomial Model	*p*-value	Odds Ratio	
Age		<0.001	0.91	0.211
MMSE		0.258	0.86	
BMI		0.331	1.04	
Group: IG–CG		0.042	1.91	
Health condition: low–high		0.140	0.36	

CG: Control Group; IG: Intervention Group; ADL: Activities of Daily Living; IADL: Instrumental Activities of Daily Living; AADL: Advanced Activities of Daily Living; MMSE: Mini-Mental Status Examination; BMI: Body Mass Index. * Results use as a starting point the level of high risk of falling, frailty, and moderate dependence and assess changes in the other categories. ** For IADL, the starting point is the moderate dependency level, which is used to analyze changes in the mild level. *** For AADL, the reference category is not autonomous and assesses the opportunity to become autonomous.

## Data Availability

The data presented in this study are available upon request from the corresponding author. The data are not publicly available because, due to the sensitive nature of the questions asked in this study, participants were assured their raw data would remain confidential and would not be shared.
